# Short-term spontaneous fluctuations of HBV DNA levels in a Senegalese population with chronic hepatitis B

**DOI:** 10.1186/s12879-015-0881-4

**Published:** 2015-03-25

**Authors:** Sarah Maylin, Jean-Marie Sire, Papa Saliou Mbaye, François Simon, Anna Sarr, Marie-Louise Evra, Fatou Fall, Jean Daveiga, Aboubakry Diallo, Jean-Marc Debonne, Loic Chartier, Muriel Vray

**Affiliations:** Laboratoire de Virologie, Hôpital Saint-Louis, AP-HP, 1 avenue Claude Vellefaux, Paris, France; Université Paris-Diderot, Paris, France; Laboratoire de Microbiologie, Centre hospitalier intercommunal de Poissy-Saint-Germain-en-Laye, Poissy, France; Department of Hepatology and Gastroenterology, Principal Hospital, Dakar, Senegal; Inserm U941, Paris, France; Department of Hepatology and Gastroenterology, Abass Ndao Hospital, Dakar, Senegal; Department of Hepatology and Gastroenterology, Saint-Jean de Dieu Hospital, Thies, Senegal; Department of Hepatology and Gastroenterology, Grand-Yoff Hospital, Dakar, Senegal; Direction des Services de Santé des Armées, Paris, France; Epidemiology Unit of Infectious Diseases, Institut Pasteur, 25 rue du Dr Roux, Paris, 75015 France; INSERM, Paris, France

**Keywords:** HBV DNA level, HBsAg quantification, Short-term spontaneous fluctuation, Chronic Hepatitis B

## Abstract

**Background:**

We evaluated the short-term spontaneous fluctuations of HBV DNA and HBsAg levels in Senegalese patients with chronic infection with hepatitis B virus and normal ALT and determined factors related to these fluctuations.

**Method:**

A total of 87 patients with persistent normal ALT values were enrolled in the study. Serum samples were obtained at three different visits, with an interval of 2 months (M0, M2, and M4), and without initiating anti HBV treatment. Levels of HBV DNA, quantitative HBsAg, ALT and AST, genotyping and viral DNA mutations were analyzed.

**Results:**

Among the 87 patients, genotype E was predominant (75%). The median HBV DNA level was 2.9 log_10_ IU/mL [2.2-3.4], 2.7 log_10_ IU/mL [2.1-3.6] and 2.7 log_10_ IU/mL [2.1-3.4] at M0, M2 and M4, respectively. The values ranged from <1.1 to 7 log_10_ IU/mL and 55 (63%) had HBV DNA fluctuations ≥ 0.5 log_10_ IU/mL between two visits. Patients in whom HBV DNA fluctuated ≥0.5 log_10_ IU/mL between M0 and M2 also had significant fluctuations between M2 and M4, while patients with stable HBV DNA between M0 and M2 showed a stable viral load between M2 and M4. The only factor found to be associated with HBV DNA fluctuations ≥ 0.5 log_10_ IU/mL was a low BMI (<21 kg/ m^2^). HBsAg levels were not correlated with HBV DNA levels.

**Conclusion:**

Sixty-three percent of the enrolled Senegalese population showed a large, short-term fluctuation of HBV DNA levels. Such fluctuations may have an impact on therapeutic management, requiring closer monitoring.

## Background

Hepatitis B virus (HBV) infection is a global epidemic, with more than 350 million of chronically infected carriers of the virus surface antigen (HBsAg) [1]. Without treatment, 15 to 40% of those with chronic HBV infection will develop cirrhosis that can potentially lead to hepatocellular carcinoma [2].

HBV is hyperendemic in Senegal, where the prevalence of chronic HBsAg carriage is about 15-20% in the general population [3]. Senegalese patients are primarily infected during early childhood, and genotypes E and A predominate in these infections. HBV infection in Senegal shows an 83% rate of precore mutations [4]. Reliable, easy-to-perform markers are needed to assess the impact of chronic HBV infection on liver diseases. Quantification of plasma HBV DNA by real-time PCR, combined with liver biopsy histo-pathological stage and biological markers of hepatocyte cytolysis level, can distinguish inactive HBsAg carriers from patients with active disease who require treatment. Patients should be considered for treatment when they have HBV DNA levels above 2000 IU/mL (3.2 log_10_ IU/mL), or serum alanine aminotransferase (ALT) levels above the upper limit of normal (ULN) and liver biopsy suggestive of significant fibrosis (≥F2) [5]. In developing countries, viral load measurement is rarely accessible. If only one measure of viral load is available, it may not reflect real viral activity. Possible fluctuations may affect the indication of treatment. However, several studies have shown long-term HBV DNA spontaneous fluctuations [6,7]. The magnitude of these changes is likely to change a treatment decision [7]. Short-term fluctuations and factors affecting them remain unknown [8].

Recently, HBsAg levels have been proposed as a marker for monitoring HBV infected patients [9,10]. HBsAg levels change over the natural course of chronic HBV infection and during antiviral therapy. Moreover, HBsAg quantification can be used to differentiate true inactive carriers with HBsAg level <1000 IU/mL from patients in remission who are likely to progress to cirrhosis [11]. The aim of this work is to describe spontaneous HBV DNA and HBsAg level fluctuations over a four month- follow-up period among Senegalese patients positive for HBsAg with normal ALT, so as to identify factors associated with these fluctuations.

## Methods

### Patient population

Patients were consecutively enrolled by private practitioners and by four public hospitals in Dakar, Senegal’s capital city, from September 2005 to April 2006. Treatment-naive patients above 18 years old, with positive HBsAg over six months, symptom-free, HIV, HCV and HDV negative, were eligible for enrolment.

Eighty-seven patients with persistent normal ALT values were prospectively monitored for determination of ALT and aspartate aminotransferase (AST), quantification of HBV DNA three times at two-month intervals (M0, M2, and M4). For those with positive HBV DNA, genotyping and mutations affecting the expression of HBeAg were performed.

### Ethical approvals

The protocol was in accordance with Declaration of Helsinki ethical guidelines and was approved by the Senegalese Health Research National Council. Patients fulfilling the inclusion criteria were enrolled after providing written and informed consent. Lamivudine was proposed to patients eligible for treatment, according to the above mentioned criteria.

### Material and methods

#### Data collection

The following data were collected [1]: general characteristics (age, sex, weight, height, known HBV infection duration, body mass index (BMI)) [2]; biological markers at the inclusion (genotype, HBeAg, HBsAg and platelets). HBV DNA, HBsAg quantification, ALT, AST, were measured three times with intervals of two months (M0, M2 and M4). ALT and AST results were expressed relative to the normal values according to the technique used (Ortho Clinical Diagnostics, Issy-les-Moulineaux, France) i.e. 52 IU/mL (females) and 72 IU/mL (males) for ALT, and 36 IU/mL (females) and 59 IU/mL (males) for AST. Variations of ALT, AST, HBsAg and HBV DNA between two visits were determined and expressed as ΔALT, ΔAST, ΔHBsAg and ΔHBV DNA, respectively.

### Biological markers

HBeAg and qualitative HBsAg were performed by an automated EIA method (Axsym, Abbott Diagnostics, Rungis, France). Biochemical parameters were determined by Vitros 250 instrument (Ortho Clinical Diagnostics, Issy-les-Moulineaux, France). Platelets were determined by Cell-Dyn 3700 (Abbott).

### Virological analyses

The HBV DNA quantification was performed using the Cobas AmpliPrep/Cobas Taqman HBV test, v1.0 assay (Roche Diagnostics, Meylan, France), with a detection threshold of 12 IU/mL (1.1 log_10_ IU /mL).

The core mutation W28 at nucleotide 1896 (precore mutation C28) and clade genotyping were determined by research DNA microarray (bioMérieux, Marcy l’Etoile, France) [12].

### HBsAg quantification

Serum HBsAg levels were retrospectively quantified using sera stored at −20 °C, which had been used for HBV DNA measurement. Architect HBsAg EIA (Abbott, Rungis, France) [13] was used with a dynamic range of 0.05-250 IU/mL. Samples with HBsAg >250 IU/mL were diluted to 1/100 to bring the value within the range of calibration [14].

### Statistical analysis

Continuous variables were expressed as median and interquartile ranges (IQR), and categorical variables were expressed as percentages. Univariate analyses were based on Fisher’s exact test for categorical variables and Mann–Whitney test for continuous variables. The HBsAg (log_10_ IU/mL) to HBV DNA (log_10_ IU/mL) ratio was assessed for all serum samples according to HBV DNA levels (≤3,]3–4],]4–5], and > 5 log_10_ IU/mL). Comparison of ratios between levels of HBV replication was analyzed with the Kruskal-Wallis non-parametric test. When a significant difference was detected, Mann–Whitney test was performed, with Bonferroni correction for multiple testing. HBsAg and HBV DNA fluctuations were measured three times at two month intervals (M0, M2 and M4). For each subject, the largest difference in HBV DNA levels between two consecutive visits was recorded and classified according to four classes: ≤ 0.5,]0.5-1][,]1-2] and >2 log_10_ IU/mL. Friedman’s test was used to compare HBsAg fluctuations between the three values. Correlation between viral load fluctuations was based on the Spearman coefficient.

All variables associated with HBV DNA fluctuations > 0.5 log_10_ IU/mL in univariate analysis (p < 0.25) were included in a backward stepwise logistic regression model. A *P* value of ≤0.05 was considered to denote statistical significance. Statistical analyses were performed using STATA software version 12.0 (Stata Corporation, College Station, TX).

## Results

### Population characteristics

The characteristics of the studied population are summarized in Table 1. The population was primarily male (72%), with a median age of 30 years and a median BMI of 21 kg/m^2^. All patients maintained normal ALT, with a median of 0.5xULN (upper limit of normal) [0.4-0.6] over the three visits. Genotype E was predominant (75%), the remaining belonging to genotype A. The median HBV DNA level was 2.9, 2.7 and 2.7 log_10_ IU/mL for the first, second and third visits, respectively (Table 2). The values ranged between <1.1 (undetectable) and 7 log_10_ IU/mL. Thirty-six subjects (41%) had at least one visit with a viral load ≥3.2 log_10_ IU/mL. Only 15 patients (17%) were above ≥3.2 log_10_IU/mL and five patients (6%) had an undetectable HBV DNA (<1.1 log_10_ IU/mL) throughout the three visits.

**Table 1:** **Characteristics of the 87 subjects**

**Variables**	**N (%)**	***Median [IQR]***
Age (years)		30 [25–38]
Gender (males)	63 (72)	
BMI *(*kg/m^2^)		21 [19-24]
- ALT(xULN*)		0.5 [0.4-0.6]
- AST (xULN*)		0.5 [0.4-0.7]
Platelets (giga/L)		194 [163–245]
**Precore (W28) mutation (n = 62)**		
- Wild type	35 (56)	
- Mutation	27 (44)	
**HBV Genotype (n = 63)**		
- A	16 (25)	
- E	47 (75)	

*ULN: upper limit of normal.

**Table 2:** **HBsAg and HBV DNA levels in the 87 subjects at M0, M2 and M4**

**Variables**	**N(%)**	**Median [IQR]**
HBV-DNA log_10_ IU/mL		
- First sample		2.9 [2.2-3.4]
- Second sample		2.7 [2.1-3.6]
- Third sample		2.7 [2.1-3.4]
∆HBV-DNA log_10_ between 2 visits		
- ≤0.5	32 (37)	
-]0.5-1]	31 (36)	
-]1-2]	16 (18)	
- >2	8 (9)	
HBV-DNA log_10_		
- <3.2 log_10_ for all the visits	36 (41)	
- ≥3.2 log_10_ for 1 or 2 visits	36 (41)	
- ≥3.2 log_10_ for all the visits	15 (17)	
HBsAg IU/mL,		
- First sample		4672 [1473–8355]
- Second sample		4546 [1749–8871]
- Third sample		3473 [1241–8765]

Ninety percent of patients had a detectable viral load at baseline (M0) and among them, two patients (5%) had a positive HBeAg. Ninety four percent of patients had at least one visit with detectable viral load, while 6% were always undetectable (<1.1 log) over all the visits.

### Viral load fluctuations

**Figure 1:**
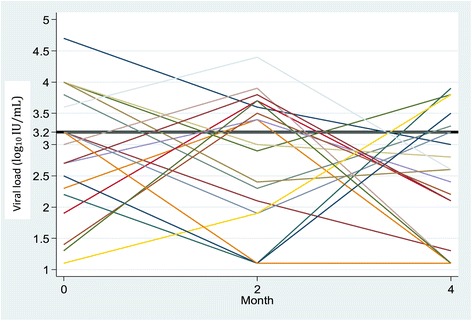
**Viral load fluctuations between M0 and M4 in patients with a change in viral load ≥ 1 log**_**10**_ **IU/mL (n = 19)) (x axis = months; y axis = viral load fluctuations (log**_**10**_ **IU/ml).**

**Figure 2:**
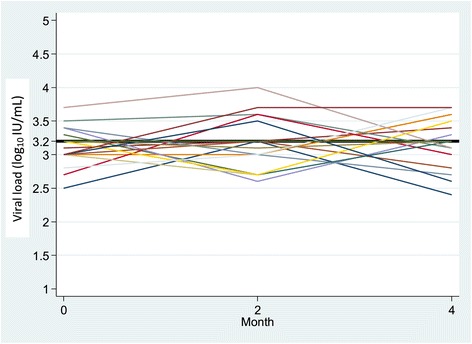
**Viral load fluctuations between M0 and M4 in patients with a change in viral load < 1 log**_**10**_ **IU/mL (n = 17) (x axis = months; y axis = viral load fluctuations with a change <1 log**_**10**_ **IU/ml).**

Sixty-three percent of subjects (n = 55) experienced a change in viral load of at least 0.5 log_10_ IU/mL between two visits. In 8 patients (9%) the difference was >2 log_10_ IU/mL (max 2.8 log_10_ of difference) (Table 2). Intra-individual changes over time in viral load based on fluctuation ranges are represented in the figures as spaghetti plots (Figures 1 and 2).

The only factor associated with a change in the viral load > 0.5 log was BMI < 21 kg/m^2^ (OR: 3.5; 95% CI, 1.3-9.0) (Table 3).

**Table 3:** **Factors associated with fluctuation of HBV DNA greater than 0.5 log**_**10**_ **IU/mL**

**Variables – N (%)**	**Patients with a change in viral load between two samples**		**p value**
	**≤0.5 log**_**10**_ **IU/mL (n = 32)**	**>0.5 log**_**10**_ **IU/mL (n = 55)**	
Gender (males)	23 (72)	40 (73)	0.93
Age ≥ 30 yrs	22 (69)	26 (47)	0.052
BMI < 21 (kg/m^2^)	10 (31)	32 (62)	0.007
Platelets (giga/L)*	188 [168–214]	209 [161–247]	0.94
∆ALT*	3 [1-8]	5 [3-9]	0.19
∆AST*	2 [1-6]	4 [2-7]	0.075
∆HBsAg* (n = 51)	924 [355–1248]	627 [218–1479]	0.99
Genotype E (n = 63)	15 (79)	32 (73)	0.60
Precore mutation C28 (n = 62)			
- Wild type	9 (50)	16 (36)	0.32
- Mutation	9 (50)	28 (64)	

* ***median [IQR].***

**Figure 3:**
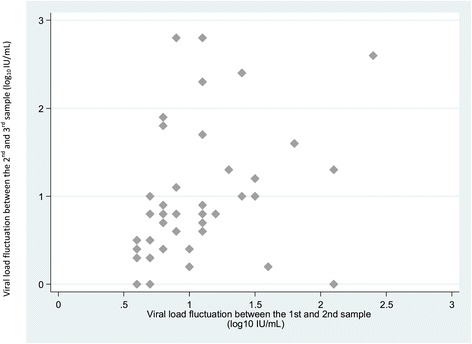
**Viral load fluctuation (log**_**10**_ **IU/mL) between the second and third samples in patients for whom viral load fluctuation between the first and second sample was >0.5 log**_**10**_ **IU/mL (x axis = viral load fluctuations between the first and second sample (M0 et M2) (log**_**10**_ **IU/mL); y axis = viral load fluctuations between the second and third sample (M2 et M4) (log**_**10**_ **IU/mL).**

**Figure 4:**
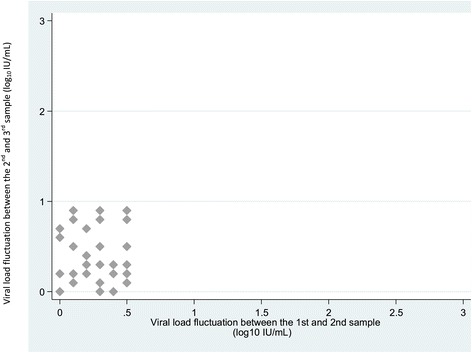
**Viral load fluctuation between the second and third samples in patients for whom viral load fluctuation between the first and second sample was ≤0.5 log**_**10**_ **IU/mL.** (x axis = viral load fluctuations between the first and second sample (M0 et M2) (log_10_ IU/mL); y axis = viral load fluctuations between the second and third sample (M2 et M4) (log_10_ IU/mL).

Patients in whom HBV DNA fluctuated >0.5 log_10_ IU/mL between M0 and M2 also showed significant fluctuation between M2 and M4 (positive correlation r = 0.34, p = 0.005) (Figure 3), while patients with stable HBV DNA between M0 and M2 showed a stable viral load between M2 and M4 (Figure 4). Such significant fluctuations were only observed in patients with genotype E viruses (positive correlation r = 0.42, p = 0.05). For 45% of the patients, the largest variation observed between two samples was between the second and third samples.

### HBsAg fluctuations

The values of HBsAg differed significantly between the three visits (p = 0.03), mainly due to the difference observed between the second and third samples, with respective medians of 4546 and 3473 IU/mL (Table 2).

We did not find a link between HBsAg fluctuations and a change in the viral load > 0.5 log_10_ IU/mL (Table 3). There is no significant difference in HBsAg levels, regardless of the genotype (A/E) or the presence of a precore mutation C28 (Table 4). The median HBsAg level in patients with HBeAg negative was 6612 [1879–9260], 6228 [1905–9089) and 5393 [2269–9883] IU/mL for the first, second and third samples, respectively. The HBsAg level was 7584 and 8765 IU/mL for the second and third samples for the sole patient with positive HBeAg.

**Table 4:** **Fluctuations of HBsAg levels**

**Variables – Median (IQR)**	HBsAg levels* (1 ^**st**^ **sample)**	**p**	HBsAg levels* (2 ^**nd**^ **sample)**	**p**	HBsAg levels* (3 ^**rd**^ **sample)**	**p**
Genotype (n = 43)		0.37		0.30		0.33
- E	5648 (1914–10404)		5545 (2571–9876)		4486 (2611–9624)	
- A	6429 (618–9134)		6228 (635–8871)		4943 (610–9896)	
Precore mutation C28 in negative HBeAg patients		0.26		0.56		0.56
Wild type (n = 13)	7058 (1473–8355)		6228 (1556–8871)		4943 (1241–9883)	
Mutation + (n = 14)	6906 (2964–12711)		7954 (3170–10389)		6874 (2952–10703)	

*IU/mL.

### HBsAg/HBV DNA ratio

**Figure 5:**
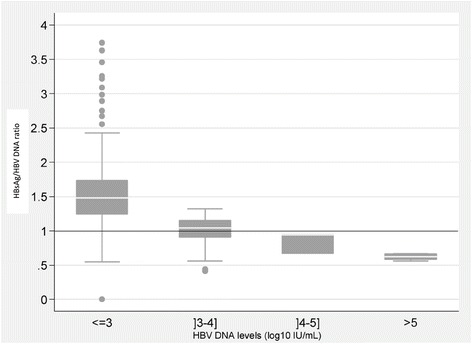
**Plots of log**_**10**_ **IU/ml HBsAg /HBV-DNA ratio by levels of HBV replication.** (all samples) (x axis = BV DNA levels (log_10_ IU/mL); y axis = HBsAg /HBV-DNA ratio).

The median HBsAg (log_10_ IU/mL) to HBV DNA (log_10_ IU/mL) ratio was significantly higher in samples with HBV DNA values ≤3 log_10_ IU/mL (1.48 [1.24-1.73]), compared to high HBV DNA values (1.04 [0.91-1.15] for]3-4] log_10_ IU/mL (p < 0.001), 0.94 [0.67-0.95] for]4-5] log_10_ IU/mL (p = 0.05) and 0.63 [0.58-0.67] for more than 5 log_10_ IU/mL, (p < 0.001)) (Figure 5).

## Discussion

This study highlights the frequency and magnitude of spontaneous HBV DNA fluctuations over a short period (4 months) among Senegalese patients with normal transaminases, as well as their possible impact on clinical characterization of the disease and its therapeutic management. The lack of association between these fluctuations and demographic parameters (age, gender) or biochemical (transaminases) or virological data (genotype, mutations affecting the expression of HBeAg) could be explained by a lack of power because of the small number of subjects included in the study. Low BMI < 21 kg/m^2^ was the only factor associated with HBV DNA fluctuations; patients with a lower BMI values were at higher risk of having fluctuations. One hypothesis is that HBV DNA fluctuations can reflect more efficient immune response which is more present in healthy, low BMI patients. As previously demonstrated, there is strong evidence that excess adiposity, defined by high BMI, negatively impacts immune function and host defenses in obese individuals [15].

The stability of the biochemical markers contrasts with the volatility of the HBV DNA.

The lower replicativity among HBeAg negative patients does not explain the HBV DNA variability observed in our study.

The frequency and magnitude of DNA fluctuations and their possible impact on clinical characterization of the disease and its therapeutic management must be emphasized. Guidelines recommend that to be eligible for treatment, patients must have a viral load greater than or equal to HBV DNA 3.2 log IU/mL. Only 17% of our patients constantly exceeded this threshold in the three samples tested (M0, M2 and M4). Forty-two percent of patients would have been eligible or ineligible for treatment according to the viral load value at a given time. It therefore seems necessary to measure viral load for multiple occasions before deciding to treat.

More recently, studies have reported that the management of chronic hepatitis B can be optimized with HBsAg quantification, used as a biomarker for stratifying the risk of disease progression [16] and for predicting treatment response mainly in patients receiving pegylated interferon (PEG-IFN) therapy [17].

In clinical practice, HBsAg quantification cannot replace viral load measurement. Combining both measures has been shown to be important for monitoring the natural history of the disease and treatment outcome [11]. We observed a significant reduction in the HBsAg level between the second and third visits. Consistent with other studies, we found no correlation between HBV DNA levels and HBsAg in these HBeAg-negative patients [18]. It has been shown that the HBsAg / HBV DNA ratio, which reflects the association between HBsAg production and HBV replication, increased after seroconversion without any HBsAg level modification. Confirming immune control over viral replication was the first step of immune clearance. This ratio has been shown to be higher during the low-replicative phase, compared to immune-tolerant, immune-clearance and HBeAg negative hepatitis phase and to be repeatable regardless to ethnicity or genotype [19-21]. Nevertheless, in our study, the HBsAg / HBV DNA ratio was higher in samples with low HBV DNA values than in those with high HBV DNA values, as reported by others [22]. These results suggest that the production of HBsAg is more conserved than the HBV DNA replication indicating that the association between HBsAg production and HBV DNA replication seems disconnected [23,24].

## Conclusion

Since HBeAg-negative patients with higher HBsAg levels were more susceptible to developing active disease, quantification of HBsAg in such patients merits further investigation.
